# The families SHARE project: novel insights on recruiting and engaging Black men in a community-based genomic education program

**DOI:** 10.1186/s12889-025-21853-x

**Published:** 2025-02-27

**Authors:** Julia R. Nummelin, Jama J. Brookes, Raegan A. Bishop, Calandra G. Whitted, Chiranjeev Dash, Laura M. Koehly

**Affiliations:** 1 Social Network Methods Section, National Human Genome Research Institute, Bethesda, MD USA; 2 Office of Minority Health & Health Disparities Research, Lombardi Comprehensive Cancer Center, Georgetown University, Washington, DC USA; 3 Social and Behavioral Research Branch, National Human Genome Research Institute, National Institutes of Health, Building 31, Rm B1B54, Bethesda, MD 20892-2073 USA; 4 Georgetown Lombardi Comprehensive Cancer Center, Georgetown University Medical Center, Washington, DC USA

**Keywords:** Minority health, Black communities, Genomics education, Family health history

## Abstract

**Background:**

Black Americans are disproportionately affected by a number of common complex conditions, such as cancer. Genomic tools like Family Health History (FHH) can be useful in guiding screening and behavior based on a person’s risk for these conditions. Factors such as family communication and societal norms can influence individuals’ knowledge of their FHH. Men, particularly Black men, are less likely than women to know FHH. Further, there is limited understanding of Black men’s participation in FHH dissemination, as they are often underrepresented in biomedical research. Understanding Black men’s perceptions of FHH sharing may help guide effective recruitment and retention efforts in future genomic research providing an opportunity to investigate their lack of engagement in FHH conversations.

**Aims:**

The purpose of this paper was two-fold: (1) Detail methods that were effective in recruiting and retaining Black men in community-based genomic research interventions, and (2) Evaluate the factors influencing men’s participation in FHH gathering and sharing.

**Methods:**

This one-year, mixed methods study combined qualitative community-based education programs (*n* = 12) and semi-structured interviews (*n* = 27), with quantitative survey assessing participant characteristics and sex differences (*n* = 50). Transcripts from the program were coded by separate study team members for themes and provided insights into study participants’ perceptions about FHH and their involvement in gathering and disseminating this information within their family.

**Results:**

Challenges in recruiting and retaining Black men prompted the study team to pivot recruitment strategies, including partnering with community-based organizations focused on men’s health, growing the research team to include Black men, adapting to potential participants’ time constraints, and creating opportunities to build trust. A thematic analysis of community education sessions and interviews identified five themes, including social role expectations and perceived family disconnectedness, that provide insights into potential barriers to participation. Qualitative data from participants suggests that beliefs and perceptions about the roles Black men play in health discussions within the family may influence their involvement, while community programs were seen as encouraging men to engage in these conversations.

**Conclusion:**

These lessons learned provide valuable perspectives on potential barriers to participation, which may inform future strategies that aim to engage Black men in family-oriented community education programs and genomic research.

**Supplementary Information:**

The online version contains supplementary material available at 10.1186/s12889-025-21853-x.

## Background

In the United States, communities identifying as Black/African American (henceforth referred to as “Black”) are disproportionately affected by common chronic health conditions, such as cancer, type II diabetes, and heart disease [1]. These diseases are etiologically complex, with genetic, environmental, and lifestyle factors all influencing disease risk. Genomics is an important part of healthcare, in that it can be used to tailor preventive services and enable healthful behaviors for those at increased risk. Family Health History (FHH) is a preventative health tool shaped by genetic, environmental, and lifestyle factors. FHH can be useful in integrating genomics into clinical care. However, patients must enter the clinical encounter with accurate FHH information to reap its benefits. Past research demonstrates that Black families know less about their FHH than White families due to lower rates of discussions about FHH and family networks [2–5]. What is not well understood is how to address this disparity, largely due to the underrepresentation of Black participants in public health genomics research [6, 7]. One approach to engage such populations is by initiating community-based programs that increase awareness and accessibility to genomics research.

In 2021, we launched the community education component of the Families SHARE project [8], which involved a series of educational workshops that introduced a personalized, FHH-based genomic risk assessment workbook [9, 10] to adult Black participants residing in the Washington, DC region. The innovation of Families SHARE is two-fold. First, the workbook focuses on FHH as a convenient yet powerful tool that captures disease prevalence related to genetic, environmental, and behavioral risk factors that cluster in families and has both personal and clinical utility [11]. With the workbook, individuals can easily determine if they or their family members are at increased familial risk and learn about relevant screening and risk-reducing lifestyle behavioral recommendations.

Second, the Families SHARE project is grounded in the Social Ecological Model (SEM), where we consider multiple levels of influence– individual, interpersonal, organizational, community– on individual health behavior [12]. Despite its importance, lack of accurate FHH knowledge can hinder its personal and clinical utility. The literature suggests that accurate FHH knowledge is often influenced by interpersonal factors, such as who collects and shares disease diagnoses within the family [13–15]. One of the goals of the Families SHARE community education program is to identify factors influencing FHH sharing and provide strategies for individuals to engage in these conversations.

Prior research has examined the role that sex plays in the gathering and dissemination of health information in families. This work shows that women are the primary “kin keepers,” who gather and disseminate health information in the family, whereas men tend not to participate in FHH sharing [16–18]. It is important for men to be involved in the gathering and dissemination process to increase the accurate flow of information, as men have personal and family health information to contribute. Additionally, they can reach children or relatives who may be more comfortable talking to men about their health and family risk. Perceptions of social roles are a hypothesized factor for why men are not involved in sharing health information at the same levels as women [19, 20]. Thus, to improve FHH sharing, we must first better understand men’s individual beliefs on their role in gathering and disseminating FHH information to build effective family-oriented community education programs. This is especially true for Black men, whose beliefs are underreported in public health genomics research due to challenges in effective recruitment and retention efforts [6, 7].

Potential explanations for unsuccessful engagement of Black men include distrust in research and government institutions and inability to create empowering research partnerships with Black men [21–25]. Black men have been historically mistreated within the research and medical fields, which has created distrust between the community and institutions [21, 25]. Even when lack of trust is not reported, studies are not effectively reaching Black men due to unrepresentative research teams; non-inclusive and inaccessible health jargon; and lack of men-focused community relationships [22–24]. The SEM can guide creation of effective recruitment and retention strategies by building trust at the interpersonal level, creating representative teams and inclusive recruitment materials at the organizational level, and collaborating with leaders and organizations at the community level [26].

## Aim

The current report describes lessons learned during implementation of the Families SHARE community education program, a community-based participatory research project guided by SEM principles and built upon strong community ties. We first describe distinct challenges encountered in reaching Black men compared to reaching Black women, the primary keepers of FHH, and how we addressed those challenges - strategies that may be relevant for future works aimed at recruiting such populations [16–18]. We then discuss the insights gained from our study participants about men’s specific engagement and involvement in FHH gathering and the dissemination of this information within their family. These lessons learned help us understand Black men’s perceptions of FHH sharing, which may inform future recruitment and retention strategies that aim to engage Black men in family-oriented community education programs and provide an opportunity to increase their engagement in FHH conversations.

## Methods

### Setting

Using a Community-Based Participatory Research approach, Families SHARE leveraged the relationships built between the community and Georgetown Lombardi Comprehensive Cancer Center’s Office of Minority Health and Health Disparities Research (OMH) [27]. OMH aimed to recruit Black participants residing in several under-resourced neighborhoods of Washington, DC.

### Project design

This mixed methods study combined qualitative community-based education programs and semi-structured interviews, with quantitative survey assessing participant characteristics and sex differences. A total of 12 community education program sessions were fielded over the course of one year; recruitment took place from January 2021 through December 2021 (a 12-month time period), and data collection continued through February 2022. The Families SHARE community education program sessions aimed to improve comprehension and perceived utility of a personalized FHH workbook (i.e., the Families SHARE workbook) [9, 10]. The education program sessions, conducted by phone via Zoom due to the pandemic, were one hour in length. Sessions were originally intended to have mixed-sex participation; however, initial sessions were predominantly attended by women. Following an adjustment in recruitment strategies, several men-only sessions were successfully held. Most sessions took place in the evening to accommodate participants’ schedules.

Participants were eligible if they were at least 18 years of age; able to read, write, and speak English; and willing to participate in the study. Individuals who were adopted were excluded from this FHH study. Once consented into the study, participants completed a baseline survey via phone interview with a study team member who recorded their responses in Qualtrics. Interview times ranged from twenty minutes to over one hour. Participants also provided a detailed FHH on five common, complex diseases, including heart disease, type 2 diabetes, and three types of cancer (colorectal, prostate, and breast) through a telephone interview that averaged approximately 25 min (see Supplemental Materials). This information was used to generate a personalized pedigree using Progeny [28], which was printed and mailed to participants alongside the workbook for use in determining their family risk of disease and identifying ways to reduce risk. After receiving the workbook, participants attended a community education program that provided information about how to use the Families SHARE workbook. Participants completed a follow-up survey, ranging from fifteen to fifty minutes, again via phone with responses recorded in Qualtrics and had the option to complete a semi-structured interview, which gathered more detailed information on how to modify the education programs to be more accessible to the community (see Supplemental Materials).

### Measures

#### Quantitative

Demographic characteristics were obtained through self-report and included sex, age, marital and familial status, educational attainment, race and ethnicity, and income. Adapted from the Genetic Literacy and Comprehension measure [29], health literacy was assessed by asking participants to rank a list of health-related words on a scale of not familiar (1) to completely familiar (7). Words included: behavior, exercise, risk, menopause, genetics, kidney, pedigree, algorithm, and colonoscopy.

#### Qualitative

A total of 12 Families SHARE community education sessions and 27 semi-structured interviews were conducted. As qualitative research saturation can be reached with a minimum sample of 12, the sample of 27 semi-structured interviews was deemed sufficient [30–32]. Community education sessions were facilitated by multiple study team members, who covered topics such as (1) the importance of FHH in complex disease risk, (2) how to read a pedigree, (3) how to use the disease risk algorithm within the Families SHARE workbook, and (4) health promotion behaviors that can reduce risk. Additionally, participants were prompted to share and discuss strategies for communicating family health information with their families and communities.

Optional semi-structured interviews were conducted via phone call after follow-up assessments were complete, one-on-one with a study team member. The interviewer asked guiding questions to gain feedback on the workbook and education program and explore topics of interest, such as the sharing of family health information (see Supplemental Materials). Where appropriate, the interviewer prompted participants to expand on their responses.

### Analysis plan

#### Quantitative

Differences by sex were assessed for socio-demographic characteristics and health literacy. Chi-squared tests of independence were used to assess differences in categorical demographic characteristics. A t-test for differences between independent samples was used to assess differences by sex for participant age and health literacy.

#### Qualitative

Audio recordings of the community education program sessions and semi-structured interviews were transcribed verbatim and de-identified. Qualitative data were analyzed to assess men’s engagement in and perception of FHH communication using a thematic analysis, which involved becoming thoroughly familiar with the transcripts and examining similarities and divergences in themes.

Four study team members individually read through all of the community education program and semi-structured interview transcripts for the purpose of determining common themes. Separate coders searched for important quotes and concepts relating to sex differences in health experiences at every level of the socioecological model. These selected quotes were recorded into individual Excel documents and were discussed among the study team, along with initial interpretive thoughts and ideas. Coding discrepancies were discussed and converged to achieve intercoder reliability. Concepts were then merged into a combined Excel document.

Later, two separate coders identified intertwined themes relating to recruitment and retention, kin-keeping activities, and Black men’s perceptions on their role in FHH sharing. Emergent themes were identified by grouping similar concepts by sex of participants. Overarching superordinate themes were created from the identified component themes through an iterative process. Thematic analysis guided interpretation of themes and promoted the uncovering of novel themes.

### Reflexivity

In accordance with the core practice of reflexivity applied to qualitative analysis, the first and second authors, who engaged in manuscript design and led qualitative analysis, identify as White American women. As White Americans, the first and second authors acted as “outsiders” to the sample, as they do not identify with the Black American racial group. Both authors have a background in public health and health disparities research, providing them with a theoretical foundation into the subject matter. A third author engaged in qualitative analysis and was able to serve as an “insider,” as they identify as Black American and have family members with common, complex diseases. Lastly, all authors engaging in qualitative analysis served as “outsiders,” as none have children, are outside the age range of participants, and do not identify as men. To minimize potential biases to data interpretation, the authors coded the interviews separately, using the consensus approach to conduct a thematic analysis and develop final codes.

### Initial recruitment & retention strategies

The initial recruitment goal was to engage a maximum of 120 participants in the project. The overall sample size for the Families SHARE community-based education program evaluation was calculated based on two factors: (1) education program group size and (2) sufficient power needed to evaluate differences in the key outcome measures. Due to the potential for within group dependencies, we considered the design effect when identifying the optimal sample size. We expected to complete approximately 12 education programs with, on average, 9 participants; oversampling was used to account for potential no-shows at the education program. Additionally, we focused on recruiting and retaining equal numbers of men and women, using convenience and purposive sampling. Active recruitment opportunities were limited due to COVID-19 restrictions, which impacted proposed recruitment strategies and goals. As such, initial recruitment strategies reached primarily women, pointing to a need to modify our strategies in order to reach more men.

In light of recent research indicating that successful recruitment of participants from historically marginalized communities, such as Black participants, requires the use of a variety of recruitment approaches and a high degree of adaptability, the study initially implemented five core recruitment and retention strategies [33–35]. These strategies, described in detail below, include: (1) community engagement, (2) study team makeup, (3) flexibility, (4) contact with participants, and (5) incentives.

#### Community engagement

Community engagement has been noted as critical to the successful recruitment of historically marginalized communities. Recruitment activities for this project were led by Georgetown’s Office of Minority Health and Health Disparities Research (OMH) through their ongoing community outreach efforts. Recruitment was conducted by trained and experienced study staff who work closely with community members and have expertise in community engagement, especially around cancer and women’s health. Utilizing OMH’s existing network, recruitment was initially conducted by a woman recruiter via flyers and word-of-mouth at community events like cancer survivor groups and support groups [27].

#### Study team makeup

The principal investigators (PIs) curated a strong research team that prioritized cultural humility—an approach to community engagement that involves recognizing, respecting, and being open to understanding different cultures, and emphasizes self-reflection and a growth mindset to build more inclusive communities—and diversity to promote recruitment and retention of Black participants. This team consisted of individuals who not only had extensive experience working with the study population but were also representative of the community. In order to engage with participants, research staff conducted face-to-face recruitment and retention strategies, ensuring that the race of study staff matched that of the participants.

#### Flexibility

The study team held weekly research team meetings for study coordination, highlighting cultural humility and flexibility in meeting participants’ needs. It was a priority of research staff to be available to participants. Phone calls were scheduled according to participant availability to best facilitate enrollment and some interviews were scheduled over multiple appointments; these strategies allowed the study team to account for participants’ work and family responsibilities. Research staff made accommodations for those who needed to complete their baseline survey and FHH assessment in two appointments, maximizing flexibility for participants.

#### Contact with participants

**Fig. 1:**
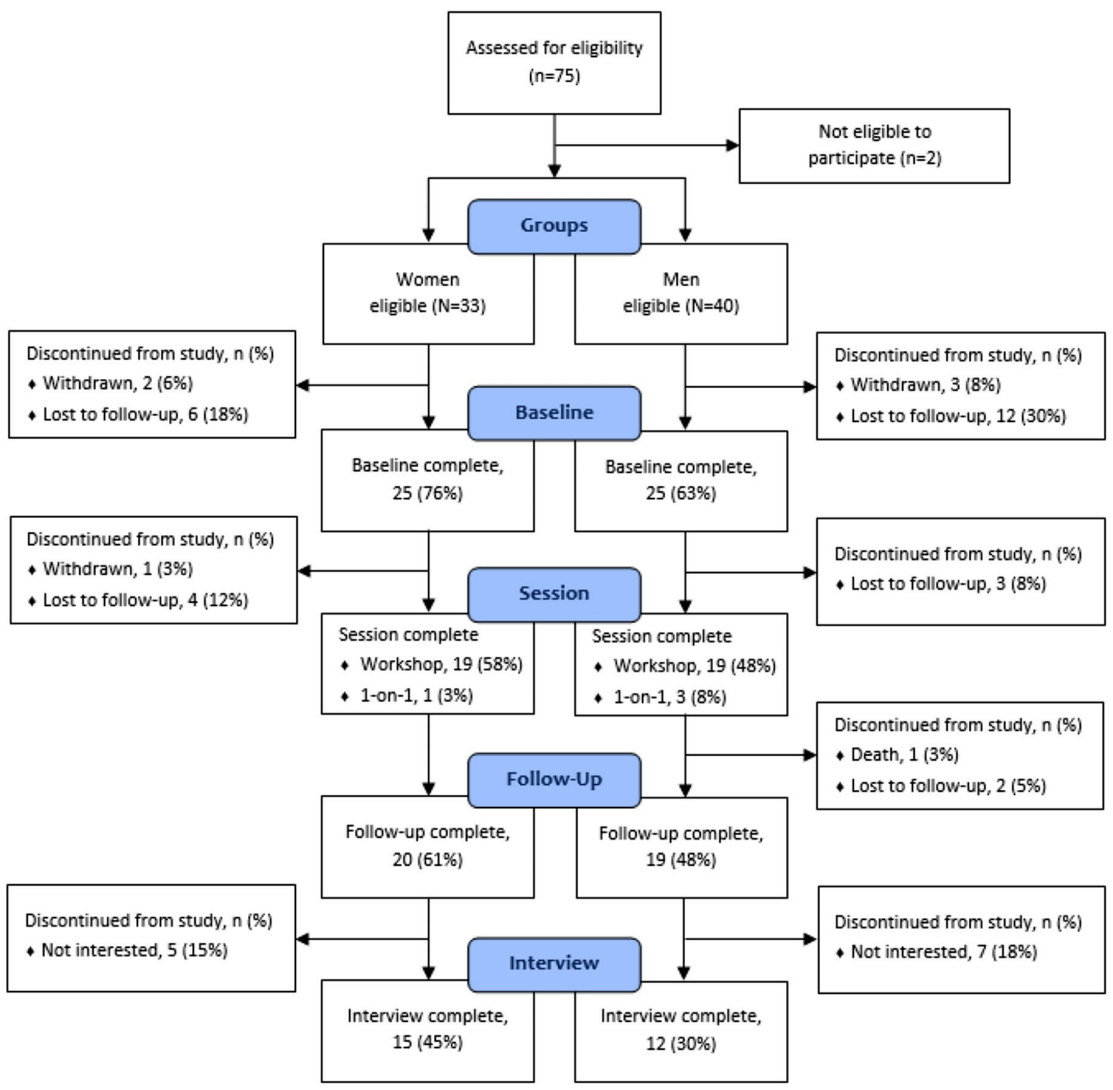
Families SHARE enrollment diagram. Notes: Diagram of recruitment and retention throughout the Families SHARE Community Education Program, stratified by sex. In total, 20 women and 19 men completed the study, with 15 women and 12 men completing an optional semi-structured interview. Percentages were calculated based on respective eligibility group (Women, *N* = 33; Men, *N* = 40)

A minimum of three contact attempts were made to reach participants. Participants received reminder phone calls prior to attending the community education program sessions, to ensure participants understood how to join the session and what materials they needed to bring to the session. Follow-up assessments were completed via telephone which minimized non-response/withdrawal. In cases where participants expressed hesitation about continuing their participation at any stage of the study, OMH study staff took proactive steps to re-engage them. This re-engagement effort had significant impact on sustaining study participation, as illustrated in Fig. 1. The participants’ responsiveness to follow-up efforts emerged as a crucial factor for retaining both Black men and women in the study and contributed to their active involvement.

#### Incentives

The use of incentives in research has been proposed as a strategy to improve recruitment and retention. Participants received a $50 gift card to a local grocery store for completing a baseline survey, community education program session, and follow-up survey. Participants who chose to complete the optional semi-structured interview received an additional $25 gift card. Additionally, participants received a copy of the Families SHARE workbook and their personalized pedigree. Upon request, the study team provided additional workbooks to participants to share within their networks.

### Recruitment & retention modifications

The research team faced several challenges recruiting Black men into study. Initial strategies from January to June 2021 resulted in recruitment of 29 women and 3 men, indicating the strategies were largely ineffective in recruiting men (see Fig. 1). Thus, study procedures were modified to recruit and engage men. Men-focused strategies were employed from July to December 2021. These strategies are described in detail below. After refocusing the recruitment strategies, 37 men and 4 women were successfully recruited into study, meeting our goal of a balanced design (see Fig. 1).

#### Community engagement

Initial community engagement strategies were successful in recruiting women. Many of these women had previously participated in OMH research, were familiar with the study team, and eager to engage with the Families SHARE project. In addition, OMH recruiters had considerable experience recruiting women for breast cancer prevention and control research. They had significantly less experience recruiting men given the OMH’s focus on women’s health. To reach more men, the study team began engaging potential participants at community events that were focused on men (e.g., veteran support groups and men’s health groups) and within specific housing communities (i.e., senior living facilities and subsidized apartment complexes).

#### Study team makeup

Matching participants with study team recruiters of the same racial background proved insufficient for recruiting Black men. The initial recruiters and program moderators who conducted the community education program were all women. To establish a more balanced team, both in terms of sex and representation, a man was brought onto the study team to act as a recruiter and another as a community education program moderator. The combination of race and sex concordance notably enhanced the engagement of participants that identified as men.

#### Flexibility

To recruit and retain men, the study team-maintained flexibility to accommodate shift work and irregular work schedules. The study team also had to build trust with participants. To do so, we developed a transfer system where recruiters who made initial contact with eligible participants introduced them to other study staff who would complete the assessments over the phone. If participants expressed concerns about their information being tracked, we implemented alternative approaches, such as dropping off study materials and incentives rather than sending them by mail. In addition, the educational topics covered in each education program session shifted to men-focused diseases. For these groups, prostate cancer was the primary topic covered, and the session was moderated by a Black man, consistent with prior work showing that men may be more inclined to participate in men-centered group sessions [36]. Finally, for those unable to attend a community education session, we conducted 1-on-1 sessions by phone that covered the same topics and content as the community sessions.

#### Contact with participants

After participants were recruited into study, research team members struggled to contact men for assessments. Specifically, among men, participants were hesitant to answer phone calls from unknown numbers from the research team or would hang up after not recognizing the caller’s voice. To address this, we increased pre-research activities (e.g., additional phone calls) to allow time for trust and rapport to build through dialogue about day-to-day activities and interests (e.g., sports), address participant needs, and exchange contact information. Relationships with participants were established with OMH staff, who then used a three-way call to introduce participants with other members of the research team. This approach significantly increased recruitment and retention among men.

## Results

### Participant characteristics

A total of 75 participants were assessed for eligibility between January and December 2021. Seventy-three (33 women and 40 men) met eligibility criteria and were consented into study, 58.4% of our recruitment goal. Of those consented into study, 50 (25 women and 25 men) completed the baseline assessment, and 39 (20 women and 19 men) completed the study (i.e., attended the community education program and completed follow-up assessments). Of those who completed the study, 27 (15 women and 12 men) also completed an optional interview.

Of those who met eligibility criteria (*n* = 73), 34 participants (13 women and 21 men) withdrew; of those who withdrew, 28 (82%) were passive withdrawals (i.e., lost to follow-up and death) and 6 (18%) were active withdrawals. Those who actively withdrew, withdrew due to lack of interest (*n* = 5) and time constraints (*n* = 1). The enrollment diagram describes participant flow through the study procedures by sex (see Fig. 1).

Table 1 describes the sample completing baseline assessment. On average, participants were 58.46 years of age, with 68% completing some post High School education and 48% reporting a household income of $20,000 or less. Only 16% (*n*=8) of the sample reported being married and 64% reported having children.

**Table 1:** Demographics

	Overall	Women	Men	*p*-value
	*n* = 50	*n* = 25	*n* = 25	
**Age (mean**,** SD)**	58.46 (12.21)	56.84 (8.45)	60.08 (15.09)	0.35
**Education Level (%)**				0.22
Less than high school diploma	4 (8)	3 (12)	1 (4)	
High School/GED	12 (24)	3 (12)	9 (36)	
Technical/Vocational	1 (2)	1 (4)	0 (0)	
Associates/Some College	18 (36)	8 (32)	10 (40)	
Bachelor’s Degree	7 (14)	5 (20)	2 (8)	
Graduate/Post Grad	8 (16)	5 (20)	3 (12)	
**Income (%)**				0.90
20,000 or Less	24 (48)	13 (52)	11 (44)	
20,001 to 35,000	2 (4)	1 (4)	1 (4)	
35,001 to 50,000	3 (6)	1 (4)	2 (8)	
50,001 to 75,000	6 (12)	4 (16)	2 (8)	
More than 75,000	6 (12)	3 (12)	3 (12)	
Prefer not to say	9 (18)	3 (12)	6 (24)	
**Marital Status (%)**				0.49
Single	31 (62)	17 (68)	14 (56)	
Married	8 (16)	2 (8)	6 (24)	
Divorced	9 (18)	5 (20)	4 (16)	
Widowed	2 (4)	1 (4)	1 (4)	
**Children (%)**				0.14
None	18 (36)	6 (24)	12 (48)	
1 or More	32 (64)	19 (76)	13 (52)	

Notes: Chi-squared tests and t-tests were run to assess any difference in demographic characteristics by sex

### Sex differences in socio-demographic characteristics and health literacy

Results are presented in Table 1. There were no significant sex differences in participant socio-demographic characteristics by sex. On average, participants reported a high degree of familiarity with the terms presented (M = 6.19, SD = 1.06). No significant differences were observed in familiarity by sex (t=-1.33; *p* =.19).

### Social role perceptions about communicating FHH with family

A thematic analysis of the community education sessions and semi-structured interviews produced five themes: (1) Social role expectations, (2) Perceived family disconnectedness, (3) Perceived lack of family responsiveness, (4) Health discussions with friends and networks outside of family, and (5) Community education programs encourage men to share. First, we assessed perceptions of and identification with kin-keeping roles by sex, and later themes helped define Black men’s perceptions about barriers and facilitators to participating in FHH conversations. The insights learned from our study participants about FHH and their involvement in gathering and disseminating this information within their families helps us understand barriers to participation that may be situated around the research goals and context. In this section, we describe those perceptions through the detailed themes and later share how they may inform ways to engage Black men in genomic research.

### Theme 1: social role expectations

The majority of participants linked the responsibility of curating and disseminating FHH information more often with women, as opposed to men. When questioned about the primary keeper of family health information, most respondents identified a woman in their family (or themselves, in the case of woman participants) rather than a man in their family. For example:

010 [W]: *And she– the one who’s left– fills me in on information because she’s the matriarch right now of the family. And she is now dealing with breast cancer. So*,* once she’s gone*,* the information is left with me; I’m the last female left on that side.*

033 [M]: *Well*,* I’m not the keeper of that information*,* my little sister is*,* so…*

Additionally, men were not only seen as disinclined to discuss information about their family’s health history but were also perceived as unwilling to share details about their personal health. For example:

055 [M]: *Well*,* most Black men period. They don’t– they’re not really going to talk about their health… I just had a friend recently who had pains in his chest. For weeks*,* he didn’t go to the doctor*,* now he’s dead because he didn’t check on the pains for his chest.*

066 [M]: *I find that the younger generation*,* there’s a difference between the boys and the girls; my nieces and nephews. The girls are more forthcoming*,* and the boys are not. So*,* I think it’s*,* you know*,* might be some machismo involved with that*,* you know. I’m strong*,* I’m good*,* you know*,* nothing’s wrong with me. But girls are more forthcoming with their issues*,* so…*

019 [W]: *And my dad -- I don’t know. He just -- like*,* he stubbed his toe*,* and he didn’t say anything. It was my little brother and my little sister that kept saying*,* “Do you smell something?” And then they looked down and saw my dad’s foot. And they rushed him to the hospital*,* but it was too late. He had to get his toe cut off. And then from the toe*,* he ended up missing a leg*,* so*,* you know.*

### Theme 2: perceived family disconnectedness

Qualitative data from the men participating in the community education program sessions and semi-structured interviews consistently revealed that barriers exist for Black men when it comes to sharing family health information. In particular, a clear trend emerged where Black men demonstrated a reluctance to share health information when they perceived a disconnect from their families and expressed concerns about privacy. Some indicated they did not engage in sharing FHH largely due to a lack of interpersonal closeness with immediate family. For example:

065 [M]: *My family’s kind of weird. Like*,* we all don’t talk like we should*,* you know what I’m saying? But*,* but the family that’s close to me that I spend more time around*,* we have conversations about various things. But*,* like*,* the distant family members*,* like we don’t talk that much*,* so those conversations never happen.*

032 [M]: *…since my mom’s passing*,* [my family’s] not close-knit.*

057 [M]: *My family keep*,* they’re too secretive. They don’t let nobody know their business*,* but they tell all their friends their business.*

031 [M]: *I get along more with my in-laws than I do with my blood relatives.…I have no history of nobody in my family except my spouse.*

030 [M]: *…in my family mostly everybody knows*,* keep their own information for themselves. And sometimes -- so like it if it’s serious they might call me and tell me.*

### Theme 3: perceived lack of family responsiveness

Despite the common belief that men do not share family health information and are not the official keepers of such knowledge within their families, our findings suggest that, from their perspective, Black men do share health information with others. However, men expressed reservations about how their messages are received by their family members. Further, it was observed that Black men do not feel as though their advice is valued by family members when attempting to discuss FHH information. For example:

060 [M]: *They are open to coming to me and everything*,* but… I give them*,* tell them things that I done read up on*,* but they still don’t go by it.*

001 [M]: *I talked to my youngest brother [about diabetes]… he said he don’t care about it… He won’t go to the doctor and see about it. He said --and so*,* he just said*,* flat out whatever happened to him is just going to happen.*

059 [M]: *I don’t have a clue as to how to talk to my kids about it because they don’t seem to be interested. And*,* you know*,* it’s not well received in my family. It’s not nothing that they really want to talk about. And*,* you know*,* so I don’t.*

### Theme 4: health discussions with friends and networks outside of family

Men who participated in this study expressed numerous obstacles hindering the sharing of family health information; however, they also shared various factors that facilitated sharing family health information. Specifically, men in the study articulated a preference for engaging in discussions about health-related topics with friends rather than family members. For example:

032 [M]: *I got a better chance of talking to my friends than my family. Because I see my friends*,* you know*,* every day in the building*,* and they got health problems and we talk about*,* you know*,* each other health problems. But I ain’t like really know [my family]. So*,* I have better–like I said*,* I have a better chance talking to my friends than my family.*

030 [M]: *Me and my friends keep most of my [family health] information.*

066 [M]: *Well*,* I think this is timely opportunity for me because I told my family at the first of the year*,* I wanted to have a discussion because I’m in the men’s health group; it was specifically for the guys. But this study gives me the opportunity to open it up to the guys as well as the women in the family.*

### Theme 5: community education programs encourage men to share FHH

Finally, it was evident that participating in community education programs like Families SHARE may be a key gateway for encouraging men to participate in sharing FHH information. The majority of the men that participated voiced that the program clarified the important role they can play in their family, empowering them to have conversations about FHH. For example:

032 [M]: *Since you brought it to my attention*,* like maybe I need to start reaching out to my people. You know*,* the ones that I can really maybe talk to and see what’s going on. You’re right. Maybe I can reach out.*

030 [M]: *Yeah*,* I think I’m going to try to finally reach out to my brothers and*,* you know*,* try to get them to sit down and listen to me. See what’s going on with them daily.*

## Discussion

In this paper, we highlight potential challenges and approaches to engage Black men in FHH interventions and broader genomic research. The purpose of this paper was two-fold. First, we detail how we modified our study procedures to engage Black men in community-based FHH interventions, implementing methods that may be useful in future intervention recruitment efforts. Second, we share insights gathered from our study participants that can inform strategies for improving Black men’s engagement in FHH communication research. Our results highlight important areas of consideration when designing community interventions that seek to engage Black men to elevate their voices through research, including recruitment strategies and materials.

Throughout the early stages of our study, we found that strategies that were successful in reaching Black women were not successful in reaching Black men. Specifically, modifications were made to community engagement practices, the composition of the study team, pre-research activities, and educational topics. Work by Yaremych and Persky [37] shows that men are more responsive to targeted recruitment attempts specific to men rather than generalized materials. Further, there is also evidence that Black men are more likely to respond to recruitment efforts that are culturally and environmentally relevant [38].

Pre-research activities that focus on relationship building to establish trust with Black men living in the community were found to be an essential part of their recruitment and retention. As Black men have endured a number of unethical practices in the name of research throughout U.S history and have an elevated level of medical mistrust [39], establishing relationships is often found to be necessary to ensure their engagement and retention in research and interventions [40–42]. The use of a multistep process was key to our success. First, we worked with community advisors to build relationships with potential study participants– a practice that can enhance development and sustainability of these new relationships [43]. Second, we also found that continued contact with participants from community-based study team members was essential to reaching and engaging Black men. While follow-up calls were initially limited to three attempts, we found that retaining Black men required more frequent and consistent contact. While requiring additional effort, the method of consistent contact can be successful in acquiring a representative sample of participants who identified as Black men [35, 44].

Reasons provided by potential participants for being hesitant to engage or choosing not to engage in the study included privacy concerns and relevance to the study participant. More specifically, in line with much of the literature on marginalized groups, we found that participants were concerned about being tracked through their participation in the study [45–47]. Participants’ privacy fears suggest the need for a certificate of confidentiality and regular restatements of study protocol confidentiality stipulations in a manner understandable by all education levels. By clearly and frequently conveying that participants will not be tracked and that their information cannot be traced back to them, we were able to build trust and assuage participant concerns. Further, the study flyers included the word “Family” which may have turned men away; our qualitative data suggests that many may have believed that the study wasn’t “for them.” As such, evaluating seemingly neutral wording and taking into consideration the complex structure of many Black Americans’ households and familial relationships is key as it may contribute to other dynamics including FHH communication.

While previous research indicates that men are less likely to engage in FHH conversations, knowledge of the underlying causes is limited [14, 18, 48]. The qualitative data from this study details men’s perceptions of the challenges they experience when attempting to gather and share FHH information within their family. There was also a general theme of men not being involved in discussions about their personal health and FHH information, however the reasoning behind the lack of sharing was complex. Largely women reported themselves and other women in their family as the keeper of this information, while men reported that they did not talk about their health with family members. Older participants also reported seeing similar differences in young adults with boys sharing less health information than girls. One potential explanation for these differences in sharing is social role expectations that begin to be reinforced at a young age; results support previous findings that women are more likely to share FHH [14, 49–51]. Interestingly, research has also indicated that masculinity can be associated with a lack of engagement in preventative health behaviors particularly among men [52, 53]. As such, it is unsurprising that men do not share when they are facing health issues and may explain why they are viewed as engaging in FHH communication less frequently. Importantly, community education programs, like Families SHARE, can empower Black men to overcome barriers when initiating and engaging in health-related conversations within their families.

We also identified themes regarding the barriers that Black men face when sharing FHH. One of the barriers men reported facing was family disconnection. These findings are consistent with prior research across multiple groups that found that family dynamics predicted health information sharing within families, with high family cohesion, openness, and adaptability associated with high levels of FHH communication [54, 55]. We also identified a theme of perceived lack of responsiveness to FHH information when they did share with others. Multiple men reported sharing information, however those they spoke with did not engage with the information as expected; as such, some men felt unheard and no longer engaged in health discussions with their family. Interestingly, men did report sharing FHH information with friends in their community. There is evidence from the literature that men are more likely to seek support outside of their nuclear family, including when discussing FHH [56]. Thus, sharing outside of their family may provide a safe resource while maintaining an appearance of masculinity within their immediate family alleviating concerns about appearing weak or needing support [57, 58]. Our findings indicate that non-familial relationships may be an avenue of intervention to encourage Black men to share their individual health concerns and FHH with family members. Taken together, these beliefs and perceptions can inform both strategies for engaging men in health research and educational programming to help Black men initiate conversations about health with their family. Without men in the conversation, families are missing important health information that has both personal and clinical utility.

### Limitations

While this study provides important insight into tailored strategies for recruiting Black men in community-based health education programs and interventions, there are several limitations to consider. COVID-19 seriously impacted study implementation as the data were collected during the height of the pandemic. The pandemic likely affected our ability to meet our recruitment goals, as well as our recruitment approach. While our originally proposed methodology consisted of in person sessions located within the community, we adapted to a phone-based implementation due to COVID-19 restrictions. As such, participant interactions were fully remote.

Since participants were fully remote, limited access to technology among the focal population became a challenge and, ultimately, a limitation due to the pandemic. Community members were unable to access Wi-Fi in public areas, such as libraries, cafes, or their housing complex common room. Thus, given that we were recruiting from a largely low-income population, many did not have access to Wi-Fi within their personal residence; indeed, a pre-implementation assessment indicated that only 5% of potential participants in our catchment area had access to private Wi-Fi. Thus, we were unable to recruit using online platforms, and largely used targeted methods by reaching out to prior research participants, posting flyers at housing complexes, and through community groups. This challenge may have reduced the generalizability of our study population because we may not have been able to reach the whole community due to the lack of internet during the pandemic. Additionally, participants may have been hesitant to participate in the online community education program if they did not have reliable internet.

Finally, some participants had concerns about being tracked or experiencing a loss of benefits due to their personal information being gathered. While we reiterated that their information would not be shared outside of our study team, several community members chose not to participate. Consequently, the representativeness of our sample may have been impacted, particularly for those with strong privacy concerns. Of note, qualitative data was gathered from study participants, not those who opted out of study participation and therefore may not reflect the views of those that did not participate.

### Recommendations and future directions

Effective engagement of communities under-represented in biomedical research requires tailoring recruitment strategies to the perspectives, beliefs, needs, and characteristics of that community. Approaches to community engagement, research team composition, adaptability and relationship building can all be tailored to the community. But, doing so, requires effective pre-research activities in the form of ongoing community outreach and engagement activities that span research, service, and community health education. Pre-research activities can identify community organizations that reach focal populations, identify characteristics of the community that need to be represented on the research team, and build relationships with community members in ways that build trust, identify needs, and gain understanding of beliefs and perspectives. Importantly, information garnered through pre-research activities can help shape messaging and program content that appeal to members of the community. For example, as previously noted, many participants in this study’s focal community did not have adequate access to internet. So, we recommend including study incentives that offer broadband internet during the duration of the study for communities who face similar barriers. Organizations have shown that tailoring research activities to the needs of the community can improve trust and engagement [59, 60].

While the goal of this report is to share lessons learned when engaging Black men in a genomics education program, our results also point to future programming that can address some of the challenges participants expressed when engaging in health discussions with their family. Many participants voiced uncertainty about how to communicate FHH information effectively within the family, across generations. Thus, interventions where they can practice these discussions may result in improved confidence. As well, many men stated that they shared their FHH and individual health information with their friends rather than their family. Developing an understanding of how those non-family connections can be leveraged to encourage health information sharing within families and improve health behaviors may be beneficial, and particularly effective within this group.

### Potential implications

Previous studies have noted that Black men have been underrepresented in biomedical research. The failure to intentionally engage this population has considerable implications, both in terms of research and population health outcomes. Within research, specific steps to recruit and engage Black men include flexibility, representation, and culturally relevant targeted recruitment materials. For example, our findings show that Black men are interested in sharing health information, but they feel unheard; building educational programs and interventions that address this concern is paramount. Importantly, in order for individuals to realize the benefits of their FHH, both in terms of personal and clinical utility, they must have accurate FHH information. Thus, ensuring that individuals are aware of the importance of FHH for their own health and those around them, and actively sharing that information within their family, is essential.

## Conclusion

The Families SHARE community education program aimed to reach Black men and women from under-resourced communities in Washington D.C. During our participant recruitment and engagement process, we encountered several challenges in reaching Black men that required modifications to our procedures. These modifications included partnering with community organizations focused on men’s health, growing our research team to include Black men, adapting to potential participants’ time constraints, and creating opportunities to build trust. As well, qualitative data from those who participated pointed to potential barriers to participation due to beliefs and perceptions about the roles Black men play in health discussions within the family. Future success in including Black men in FHH and broader genomic studies should consider the perspectives of traditional social roles within Black families and other factors such as disconnected family relationships. Importantly, community education programs, like Families SHARE, can provide more information about how to encourage and empower Black men to initiate and engage in family health-related discussions.

## Electronic supplementary material

Below is the link to the electronic supplementary material.

Supplementary Material 1

Supplementary Material 2

Supplementary Material 3

## Data Availability

The dataset used and/or analyzed for the current study are not publicly available due to privacy or ethical restrictions; they can be made available by the corresponding author on reasonable request.
