# Supplementary material for: The families SHARE project: novel insights on recruiting and engaging Black men in a community-based genomic education program
Source: BMC Public Health. 2025 Feb 27;25:805. doi: 10.1186/s12889-025-21853-x (PMC11869665; doi:10.1186/s12889-025-21853-x)
Supplement: Supplementary file 1 — Supplementary Material 1 [file 12889_2025_21853_MOESM1_ESM.docx]

**Families SHARE Semi-Structured Interview Questions**

## Community Education Program Utility and Feedback

1. Did the community education program help you understand the workbook better?
   - **Probe:** If so, what aspects of the community education program were particularly helpful?
2. What are ways to improve the community education program?
3. What aspects of the community education program would you use to help other family members learn from the Families SHARE workbook?

## Communication and Encouragement

1. When you were filling out the initial survey for us with your Family Health History details, did you talk with anyone to get health information?
   - **Probe:** If so, who did you talk with?
2. Who in your family is the most effective communicator or “keeper” of Family Health History information?
3. Did you show the workbook to anyone else in your family or others outside of your family?
   - **Probe:** If so, how did you do that? (e.g., sit down with them and “teach” them how to evaluate their risk or just give them the workbook)
4. Do you plan on showing the workbook to anyone else? (e.g., health care providers)
5. Did you and your family discuss ways to reduce your disease risk? Is this important to you or your family?
6. Are there factors due to the location of your residence that may impact communication of Family Health History?
7. Are there cultural factors specific to your family that may encourage or discourage communication about Family Health History?
