# Supplementary material for: The families SHARE project: novel insights on recruiting and engaging Black men in a community-based genomic education program
Source: BMC Public Health. 2025 Feb 27;25:805. doi: 10.1186/s12889-025-21853-x (PMC11869665; doi:10.1186/s12889-025-21853-x)
Supplement: Supplementary file 2 — Supplementary Material 2 [file 12889_2025_21853_MOESM2_ESM.docx]

**Families SHARE Family Health History Survey**

These last questions are now about your family. When responding, please include both living and deceased family members. Please include half and full blood relatives only. [Explain half and full blood relations if needed]. Do not include adopted relatives or step relatives. I will ask which of your family members, if any, are half relatives at the end of this section.

1. Has any male in your biological family (e.g. grandfather, father, uncle, brother, son) ever been diagnosed with breast cancer?


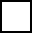
 _1_ Yes
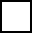
 _2_ No

So that you can identify family members on the family tree that we will send you as part of the Families SHARE workbook, I will be asking you for each family member’s first name and whether or not they have been diagnosed with colorectal cancer, breast cancer, prostate cancer, heart disease, and diabetes; given the current pandemic, we are also interested in whether any of your family members have been diagnosed with COVID-19. And, if diagnosed, their age of diagnosis.

1. What is your mother’s first name?

2a. Has she ever been diagnosed with CRC, BC, DIAB, HD, or COVID-19?
2b. If yes, at what age was she diagnosed? [Record age in years]

2c. Is she still living? [If No/DK skip 2d, 2e, 2f, 2g, 2h]


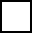
 _1_ Yes
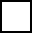
 _2_ No
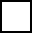
 _3_ Don’t know 2d. How old is she?

2e. Compared to where you live, where does she live?


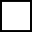

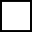

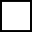
_1_ Same house _2_ Same neighborhood _3_ Same city _4_ Same state/district


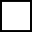

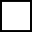

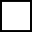
 _5_ Another state _6_ Country _7_ Don’t know

2f. In the past month, how often have you been in contact with her (either in person, by phone, or by email)?


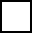

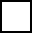
_1_ Daily or almost daily _2_ 1 to 3 times per week _3_ 1 to 3 times per month


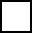
_4_ Less than once a month _5_ Don’t know

2g. If you did have contact with her in the past month, how many times did you talk to her about your health, or your family’s health?

_1_ Daily or almost daily
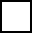
 _2_ 1 to 3 times per week
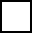
 _3_ 1 to 3 times per month


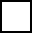

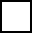
_4_ Less than once a month _5_ N/A _6_ Don’t know

2h. If you did have contact with her in the past month, how many times did she encourage you to be healthy (for example eat healthy, exercise, stop smoking, drink less alcohol, or visit a medical practitioner)?


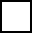

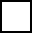
_1_ Daily or almost daily _2_ 1 to 3 times per week _3_ 1 to 3 times per month


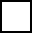

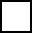
_4_ Less than once a month _5_ N/A _6_ Don’t know

1. Thinking about your mother’s relatives, what is the first name of your mother’s mother (your maternal grandmother)?

3a. Has she ever been diagnosed with CRC, BC, DIAB, HD, or COVID-19?

3b. If yes, at what age was she diagnosed? [Record age in years]

3c. Is she still living? [If No/DK skip 3d, 3e, 3f, 3g, 3h]


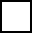
 _1_ Yes
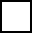
 _2_ No
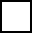
 _3_ Don’t know 3d. How old is she?

3e. Compared to where you live, where does she live?


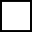

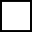

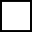
_1_ Same house _2_ Same neighborhood _3_ Same city _4_ Same state/district


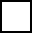

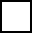

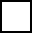
 _5_ Another state _6_ Country _7_ Don’t know

3f. In the past month, how often have you been in contact with her (either in person, by phone, or by email)?


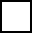

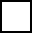
_1_ Daily or almost daily _2_ 1 to 3 times per week _3_ 1 to 3 times per month


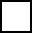
_4_ Less than once a month _5_ Don’t know

3g. In the past month, how often have you talked to her about your health, or your families’ health?


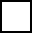

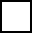

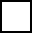

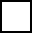
_1_ Often _2_ Sometimes _3_ Rarely _4_ Never _5_ Don’t know

3h. In the past month, how often has she encouraged you to be healthy (for example eat healthy, exercise, stop smoking, drink less alcohol, or visit a medical practitioner)?


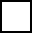

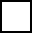

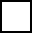

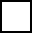
 _1_ Often _2_ Sometimes _3_ Rarely _4_ Never _5_ Don’t know

1. What is the first name of your mother’s father (your maternal grandfather)?

[Repeat items a though h, add Prostate Cancer (PC) for male family members]

1. Thinking about your mother’s siblings, what are the first names of your mother’s sisters (your maternal aunts)? [Repeat items a though h]

[REPEAT FOR EACH SISTER]

1. What are the first names of your mother’s brothers (your maternal uncles)?
   [Repeat items a though h]

[REPEAT FOR EACH BROTHER]

1. Now thinking about your father and his relatives, what is your father’s first name?
   [Repeat items a though h]
2. What is the first name of your father’s mother (your paternal grandmother)?
   [Repeat items a though h]
3. What is the first name of your father’s father (your paternal grandfather)?
   [Repeat items a though h]
4. Thinking about your father’s siblings, what are the first names of your father’s sisters (your paternal aunts)? [Repeat items a though h]

[REPEAT FOR EACH SISTER]

1. What are the first names of your father’s brothers (your paternal uncles)?
   [Repeat items a though h]

[REPEAT FOR EACH BROTHER]

1. Now thinking about your siblings, what are the first names of your sisters?
   [Repeat items a though h]

[REPEAT FOR EACH SISTER]

1. What are the first names of your brothers?
   [Repeat items a though h]

[REPEAT FOR EACH BROTHER]

1. Now thinking about your nieces and nephews, what are the names of your sister [NAME OF SISTER]’s children? 14a. Is [NAME] male or female?

[Repeat previous items a though h]

# [REPEAT FOR EACH CHILD OF EACH SISTER]

1. What are the names of your brother [NAME OF BROTHER]’s children? 15a. Is [NAME] male or female?

[Repeat previous items a though h]

# [REPEAT FOR EACH CHILD OF EACH SISTER]

1. And finally, thinking about your children, what are the first names of your daughters?
   [Repeat previous items a though h]

[REPEAT FOR EACH DAUGHTER]

1. What are the first names of your sons? [Repeat previous items a though h] [REPEAT FOR EACH SON]
2. Were any of these family members half blood relatives?
   18a. If yes, which relatives?

I just asked you to name all of your first- and second-degree relatives so that I could put together your family health history tree.

Now we are going to add to that list anyone else that you consider to be an important person in your life. The individuals don’t have to be family members, but they should be living. They can be family, friends, neighbors, coworkers, or members of your community.

Please start by naming the people that you feel very close to and that are the most important in your life. We want to protect the privacy of these people, so do not give me their full name – just their first name or you can make up a nickname for them.

[Enumeration of social network members. Allow them to list as few or many names as they like, up to a maximum of 10.]

Name 1

Name 2 Name 3 etc.

[For each nominated social network member, ask items A through H].

- 1.
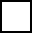
Is [NAME] male or female?
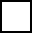
_1_ Male
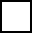
 _2_  Female _3_ Other
  2. What is [NAME]’s age?
  3.
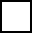

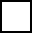
What is [NAME]’s relationships to you?


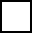

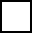
 _1_ Spouse/Partner _2_ Girlfriend/boyfriend _3_  Friend _4_ Other (specify)

- 1. [If “Other”], Is [NAME] biologically related to you?


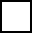

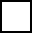

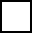
 _1_ Yes _2_ No _3_  Don’t know

2e. Compared to where you live, where does he/she live?


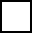

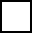

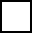
_1_ Same house _2_ Same neighborhood _3_ Same city _4_ Same state/district


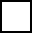

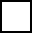

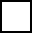
 _5_ Another state _6_ Country _7_ Don’t know

2f. In the past month, how often have you been in contact with him/her (either in person, by phone, or by email)?


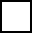

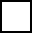
_1_ Daily or almost daily _2_ 1 to 3 times per week _3_  1 to 3 times per month


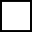
_4_ Less than once a month _5_ Don’t know

2g. If you did have contact with him/her in the past month, how many times did you talk to him/her about your health, or your family’s health?


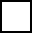

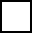
_1_ Daily or almost daily _2_ 1 to 3 times per week _3_ 1 to 3 times per month


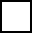

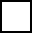
_4_ Less than once a month _5_ N/A _6_ Don’t know

2h. If you did have contact with him/her in the past month, how many times did he/she encourage you to be healthy (for example eat healthy, exercise, stop smoking, drink less alcohol, or visit a medical practitioner)?


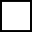

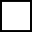
_1_ Daily or almost daily _2_ 1 to 3 times per week _3_ 1 to 3 times per month


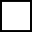

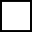
_4_ Less than once a month _5_ N/A _6_ Don’t know
